# Case report: Right coronary artery rupture—A rare complication of cardiopulmonary resuscitation

**DOI:** 10.3389/fcvm.2022.1042593

**Published:** 2022-11-07

**Authors:** Chenchen Ai, Xiaobo Lv, Xuehong Qi, Hanbo Tang

**Affiliations:** Cardiovascular Center, Gansu Provincial Maternity and Child-Care Hospital, Lanzhou, China

**Keywords:** cardiovascular injury, coronary artery rupture, hemorrhagic shock, chest compression, cardiopulmonary resuscitation

## Abstract

An 8-month-old female experienced a life-threatening right coronary artery rupture resulting from cardiopulmonary resuscitation (CPR) 1 week after corrective surgery for Tetralogy of Fallot (TOF). Emergency exploratory thoracotomy was performed due to uncorrectable hemorrhagic shock. During exploration, active bleeding was detected in the anterior branch of the right ventricular coronary artery. After the repair, the patient's condition improved. Coronary artery rupture is an extremely rare complication of CPR. Here, we present a case that provides new reflections and warnings to clinicians.

## Introduction

Effective chest compression is the key to successful cardiopulmonary resuscitation (CPR). Reports of cardiovascular injury secondary to chest compression are limited. Among these, coronary artery lacerations have rarely been reported. Owing to the absence of awareness of this type of complication, CPR-associated coronary injury may not be identified in a timely fashion. In particular, in this case, we present that some misleading information inhibited accurate judgement of the condition in time. Coronary artery rupture has a high risk of mortality and, once identified, timely surgical intervention is warranted. Here, we describe a case of rupture of the right coronary artery secondary to CPR in an infant patient who underwent corrective surgery for Tetralogy of Fallot (TOF).

## Case description

An 8-month-old female patient with TOF was admitted to our heart center. The patient underwent corrective surgery for TOF after completion of the relevant examination, and written informed consent was obtained from parents. The procedure was performed smoothly. Postoperatively, a bedside transthoracic echocardiogram demonstrated that the patient's cardiac function had improved noticeably. Owing to the small amount of drainage liquid, the pericardial drainage tube was removed on postoperative day three. However, the patient developed a pulmonary infection postoperatively, thus the tracheal intubation was left in place. Chest radiography indicated bilateral pleural effusion; therefore, closed thoracic drainage was performed. Light yellow clear fluid was observed in the left drainage tube, and light bloody clear fluid was observed in the right drainage tube. Laboratory examination of the pleural effusion suggested an exudative pleural effusion.

**Figure 1:**
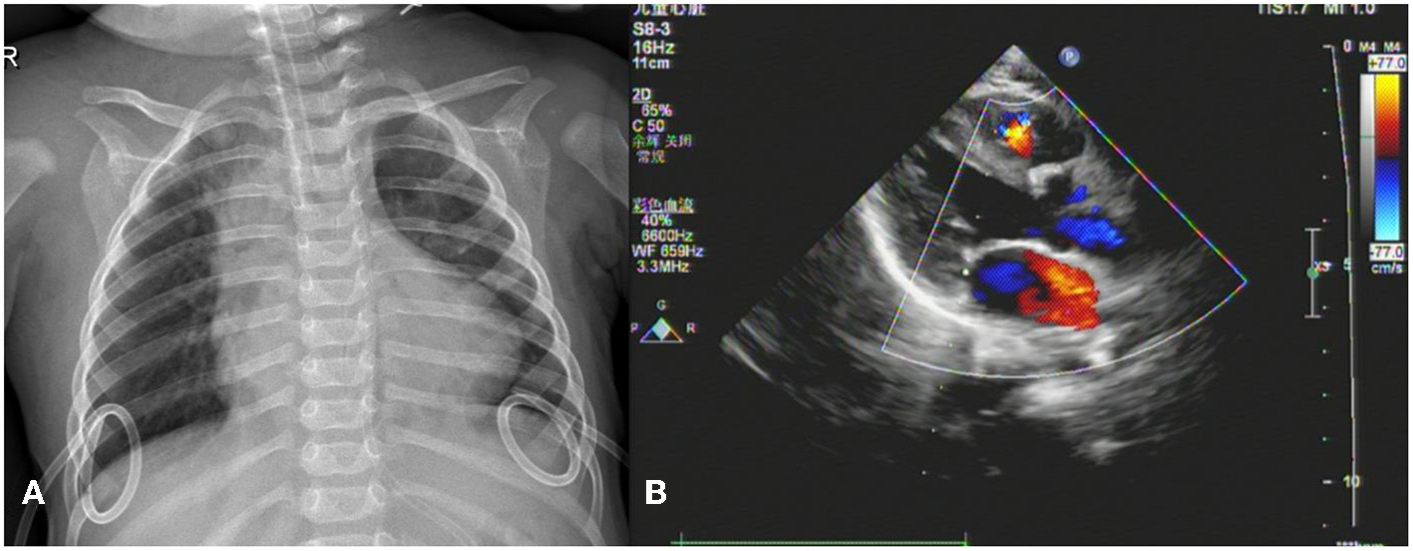
Imaging findings. **(A)** Chest X-ray shows bilateral drainage tubes and no obvious signs of pleural effusion. **(B)** No signs of pericardial tamponade or pericardial effusion on echocardiogram in left ventricular long-axis view.

**Figure 2:**
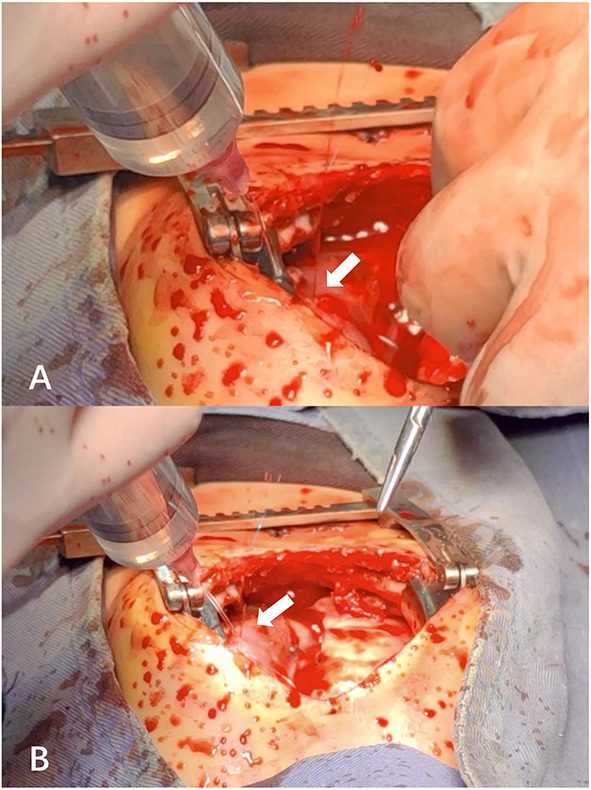
**(A,B)** Active bleeding from the anterior right ventricular branch (a branch of the right coronary artery) observed at different angles in the same operative field during exploratory thoracotomy.

**Figure 3:**
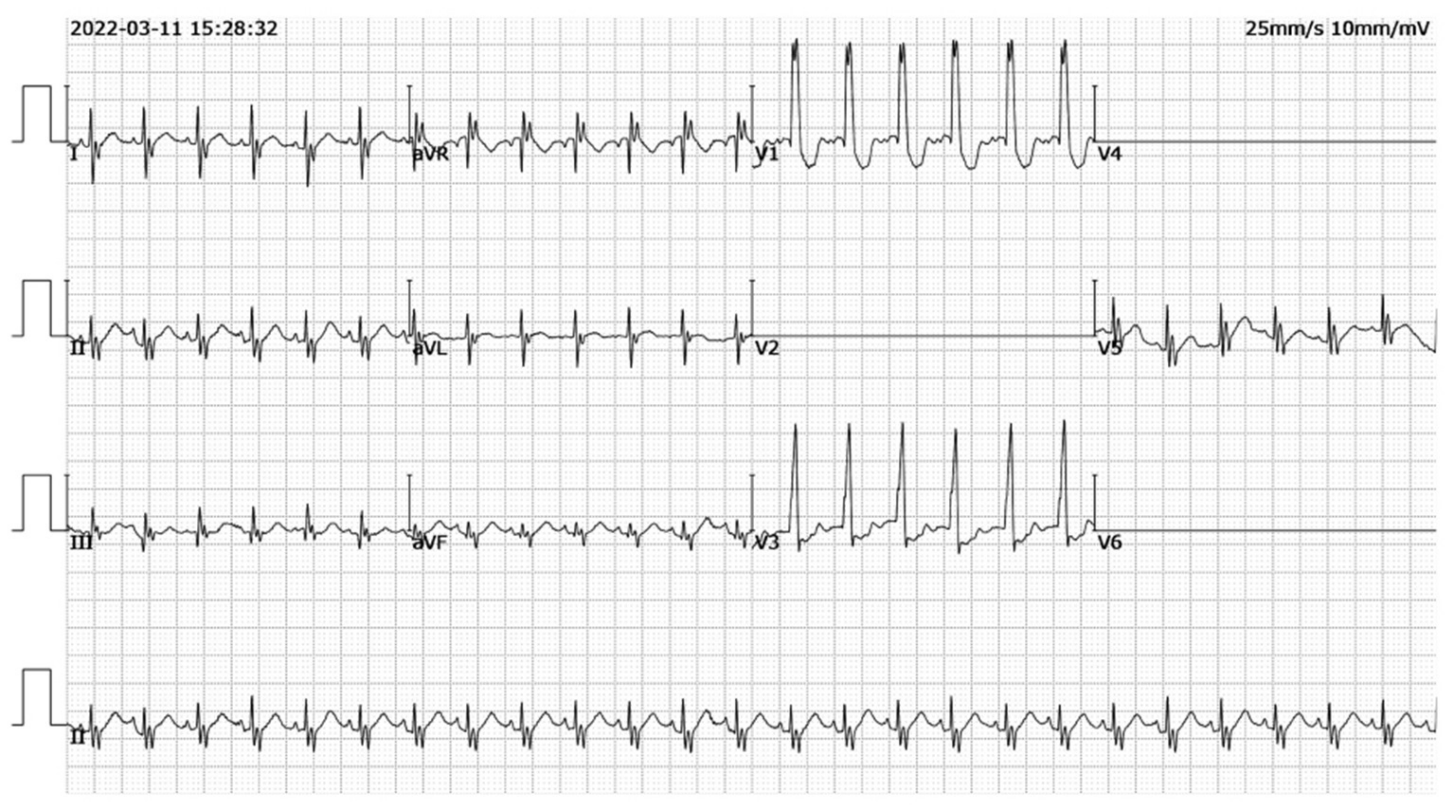
Postoperative electrocardiogram findings. No significant ST-T changes on postoperative electrocardiogram.

Thereafter, the vital signs were stable. There was no fever and the infection indicators were not high. The infant's circulatory and respiratory conditions were stable, and tracheal intubation was expected to be removed if there was no change in condition. An acute alteration occurred in the patient's condition on postoperative day seven after chest radiography. The patient developed an unstable condition with a reduced heart rate, hypotension, and hypoxemia that progressed rapidly to cardiac arrest. Effective CPR was initiated and the possible causes of cardiac arrest were evaluated. Unplanned extubation was identified as the most probable cause of cardiac arrest. After 10 min uninterrupted resuscitation, spontaneous respiratory and normal circulation returned. However, clinical observation revealed that the pre-existing thoracic drainage tube on the right side continuously drained hemorrhagic fluid, which was complicated by decreasing hemoglobin and blood pressure. The patient presented with progressive anemia and hypovolemic shock. After aggressive fluid resuscitation, hemorrhagic shock persisted. A bedside ultrasound was performed, and pericardial tamponade and effusion were not found. In addition, no significant pleural effusion was observed on repeat chest radiography (Figure 1). Therefore, emergency exploratory thoracotomy was required, given that intercostal vessel injury or intrathoracic injury during the rescue process might be possible reasons for persistent thoracic cavity bleeding. After informed consent was obtained from the immediate family, exploratory surgery was performed. The chest was entered through the original median sternal incision, and a small number of blood clots and fresh bleeding were observed in the mediastinum. After cleaning the accumulated blood, exploration continued to reveal active bleeding in the anterior branch of the right ventricular coronary artery which had a half a millimeter long tear (Figure 2). Because the tear was small, we repaired it directly using a 7-0 Prolene suture. The bleeding stopped immediately, and the patient's condition gradually stabilized. In addition, a 1 cm tear was found in the right pleura that communicated with the anterior mediastinum. Opening of the right pleura and continued exploration of the right thoracic cavity revealed no active bleeding. During 30 min observation, no ST segment changes were observed on electrocardiography. After sternal closure, the patient was returned to the cardiac intensive care unit (CCU). The patient's postoperative ECG showed no significant ST-T changes (Figure 3). The patient subsequently recovered successfully and was extubated on day three after exploratory thoracotomy and coronary repair surgery, transferred out of the CCU on day six, and discharged on day 15. At the follow-up 3 months after discharge, the patient's cardiac function had recovered well and blood indicators were normal.

## Discussion

High-quality external chest compression is the cornerstone of successful CPR. In some circumstances, this urgent medical process is accompanied by iatrogenic injuries, such as closed injuries or fractures of the chest and abdomen. Rib and sternal fractures are the most common complications of chest compression (1). However, vital internal organs, including the heart and vessels, can also be injured during this medical maneuver (2, 3).

Coronary artery rupture is often closely linked to percutaneous coronary intervention (PCI). It is also associated with macrovascular diseases such as dissection and artery aneurysms (4). However, coronary artery rupture as a complication of CPR has rarely been reported. To date, published data on CPR-associated cardiovascular injuries have been limited. Miller et al. reported an atrial rupture secondary to standard CPR, confirmed by echocardiography, and performed bedside thoracotomy to repair the breakage (5). In another study, a left coronary artery perforation was identified as a complication of CPR in a middle-aged man who had a cardiac arrest following laparoscopic inguinal herniorrhaphy (6). Early recognition and prompt therapy are crucial for CPR-associated cardiac injury, which has significant mortality, to avoid catastrophic consequences.

In the case presented here, where a female infant experienced hemorrhagic shock due to right coronary artery rupture secondary to standard manual chest compression, emergency exploratory thoracotomy was required for diagnosis. Ultimately, the patient's urgent condition was successfully relieved by prompt surgical intervention. This rare case provides unique insights regarding the cardiovascular complications of CPR. First, the strategy of chest compression in the pediatric age group differs from adults, emphasizing the “two-thumbs” encircling technique to reduce the incidence of injury to infants. Consequently, the intensity and energy of chest compression is much lower than in adults. In our case, we performed a standard CPR procedure according to the latest pediatric CPR guidelines (7). Regardless, CPR-associated cardiovascular injuries occurred in this case. Second, in regard to sternal closure in cardiac surgery, surgeons typically partially suture the pericardium to provide a protective effect. We sutured sections of the pericardium in the patient, but the post-sutured pericardium failed to completely cover the region of coronary artery distribution, which left some vessels unprotected, likely contributing to the occurrence of injuries. In addition, the tear on the right pleura caused communication between the anterior mediastinum and thoracic cavity, which was the direct reason for thoracic hemorrhage. Owing to continuous drainage of the right chest drainage tube, no obvious signs of pleural effusion were observed on chest radiography. In addition, the change in the patient's condition occurred on day seven after TOF corrective surgery, when a slight adhesion formed in the pericardial cavity, which was one of the reasons for the absence of pericardial effusion.

Early detection and evaluation of the condition of patients with CPR-related coronary artery injury are essential for effective intervention. Bedside ultrasound is a non-invasive imaging examination that aids in the assessment and diagnosis of critical patients. The main signs of coronary artery rupture on echocardiography are pericardial effusion and tamponade. However, in this case, echocardiography did not provide valuable clues for a definitive diagnosis. It also inhibited our early judgement that the source of bleeding originated from the heart vessels. Volume evaluation and fluid resuscitation are critical for hypovolemic shock. Once anemia refractory to transfusion is verified, the cause of hemorrhage must be identified as quickly as possible. In the context of an unknown etiology, prompt surgical exploration merits consideration. In our case, the solution to the critical situation of the infant benefited from a timely exploratory thoracotomy.

## Conclusion

In conclusion, cardiovascular injuries after external chest compression are rare in clinical practice. In particular, rupture of the right coronary artery is an exceptionally rare complication of CPR. Our case provides insights for clinicians based on actual practice regarding when this rare complication may occur and what clinicians can monitor to avoid fatal consequences from this rare occurrence.

## Data availability statement

The original contributions presented in the study are included in the article/Supplementary material, further inquiries can be directed to the corresponding author/s.

## Ethics statement

Written informed consent was obtained from the minor's legal guardian for the publication of any potentially identifiable images or data included in this article.

## Author contributions

CA drafted this manuscript. XL prepared the figures. XQ collected the data. HT revised the manuscript. All authors read and approved the manuscript.

## Funding

This work was supported by the Lanzhou Science and Technology Development Guiding Plan Project Grant Number: 2019-ZD-126.

## Conflict of interest

The authors declare that the research was conducted in the absence of any commercial or financial relationships that could be construed as a potential conflict of interest.

## Publisher's note

All claims expressed in this article are solely those of the authors and do not necessarily represent those of their affiliated organizations, or those of the publisher, the editors and the reviewers. Any product that may be evaluated in this article, or claim that may be made by its manufacturer, is not guaranteed or endorsed by the publisher.
